# DO END-OF-LIFE OUTCOMES DIFFER BY ASSISTED LIVING MEMORY-CARE DESIGNATION?

**DOI:** 10.1093/geroni/igae098.1661

**Published:** 2024-12-31

**Authors:** Xiao (Joyce) Wang, Porita Cornell, Emmanuelle Belanger, Kali Thomas

**Affiliations:** Brown University, Providence, Rhode Island, United States; University of Melbourne, Melbourne, Victoria, Australia; Brown University, Providence, Rhode Island, United States; Johns Hopkins University, Baltimore, Maryland, United States

## Abstract

Residential care/assisted living (RC/AL) is an increasingly common place of end-of-life care for persons with Alzheimer’s disease and related dementia (ADRD). Understanding how end-of-life outcomes differ by memory care among residents with ADRD could facilitate aging/dying in place for this population. The objective of this paper is to examine if end-of-life outcomes (i.e. mortality, hospice use, number of days receiving hospice in the last month of life) differ between residents with ADRD who moved to RC/ALs with specialized memory care service (memory-care RC/AL), compared with residents with ADRD who moved to RC/AL without memory care (general RC/AL). We created a prospective cohort of 15,152 fee-for-service Medicare beneficiaries with ADRD who moved to large RC/AL (>=25 beds) between 2016 and 2018. We used inverse probability treatment weighting to account for observable differences between memory-care and general RC/AL residents. Two-part models estimated the difference by memory care in the number of days receiving hospice in the last months of life among decedents. The adjusted difference in mortality rate was 1.3 percentage points higher in memory care (p=0.04). Additionally, memory-care residents had a higher rate of hospice use (1.4 percentage points, p=0.01). Decedents in memory-care RC/AL spent about 1.4 more days receiving hospice care in the last month of life (p=0.02). The higher mortality rate and higher rate of hospice use in memory care suggest that memory-care RC/AL may attract residents closer to the end of life and/or promote hospice use at the end of life.

